# Video assisted thoracic surgery in children

**DOI:** 10.4103/0972-9941.38910

**Published:** 2007

**Authors:** Rasik Shah, A Suyodhan Reddy, Nitin P Dhende

**Affiliations:** Department of Pediatric Surgery, Sir J J Hospital and Grant Medical College, Mumbai, India

**Keywords:** Thoracoscopy, video assisted thoracic surgery, children

## Abstract

Thoracoscopic surgery, i.e., video assisted thoracic surgery (VATS) has been in use in children for last 98 years. Its use initially was restricted to the diagnostic purposes. However, with the improvement in the optics, better understanding of the physiology with CO2 insufflation, better capabilities in achieving the single lung ventilation and newer vessel sealing devices have rapidly expanded the spectrum of the indication of VATS. At present many complex lung resections, excision of mediastinal tumors are performed by VATS in the experienced centre. The VATS has become the standard of care in empyema, lung biopsy, Mediastinal Lymphnode biopsy, repair of diaphragmatic hernia, etc. The article discusses the indications of VATS, techniques to achieve the selective ventilation and surgical steps in the different surgical conditions in children.

## INTRODUCTION

The technique of thoracoscopy was first described in the early 20^th^ century and its use in children was applied by the mid 1970s. In 1910, a Swedish internist, Hans Jacobaeus, reported the use of Nitze's lighted cystoscope in the pleural space to divide adhesions. This was performed under local anesthesia using a heated wire to divide the adhesions. This intrapleural pneumolysis was quite successful in achieving pulmonary collapse, with relatively low morbidity and mortality. Soon the technique was adopted in Europe and in the USA.[1]

In 1921, Jacobaeus reported use of this technique to diagnose benign and malignant conditions of the pleura and lung parenchyma. The rapid advances in the chemotherapy for tuberculosis diminished the role of surgery, for lung and pleural disorders. The popularity of the technique was then revived in the 1970s, when it was found to be an accurate method for obtaining lung biopsies for diagnosing Pneumocystis carinii pneumonia in immunocompromised children. Rogers was the earliest advocate of this technique for children in North America in 1976.[2] The introduction of laparoscopic cholecystectomy in 1988 led to an explosive increase in the use of minimal access surgery for different conditions. There has been a true rebirth of thoracoscopy in the last fifteen years and it is now currently accepted and used by an increasing number of practicing pediatric surgeons for a variety of indications.

### Indications for Thoracoscopy

VATS can be performed for the following conditions:

- **Diagnostic:**
  - Lung biopsy and wedge resection
    - Interstitial lung disease (ILD)
    - Biopsy of pulmonary nodule
  - Metastatic disease
  - Inflammatory disease
  - Biopsy and resection of mediastinal lesions
    - Lymphoma
    - Thymic and thyroid lesions
    - Cystic hygromas
    - Foregut duplications
    - Ganglioneuromas and neuroblastomas
- **Therapeutic:**
  - Pleural conditions
    - Spontaneous pneumothorax
    - Empyema Thoracis (ET)
    - Chylothorax
  - Extensive pulmonary resections, including segmentectomy and lobectomy for
    - Infectious diseases
    - Cavitatory lesions
    - Bullous disease
    - Sequestrations
    - Lobar pneumonia
    - Congenital adenomatoid malformations
    - Neoplasms
  - Other advanced intrathoracic procedures
    - Patent ductus arteriosus (PDA) closure
    - Division of Ligamentum in the Vascular Ring
    - Repair of hiatus hernia and other diaphragmatic defects
    - Esophageal myotomy for achalasia
    - Anterior spinal fusion
    - Repair of esophageal atresia
    - Excision of benign esophageal tumors
    - Repair of pectus excavatum
    - Sympathectomy
    - Pericardial window for pericardial effusion
    - Trans-diaphragmatic adrenal surgery

Contraindications:

- Late stages of empyema after the development of fibrosis with severe crowding of ribs (without any obvious space between ribs)
- Densely adherent tumor infiltrating chest wall

### Preoperative preparation

Most of the intrathoracic lesions require routine roentgenograms as well as CT scans. The CT scan defines the extent of disease precisely and helps in planning of surgery. Some procedures needs the specific measures preoperatively and are mentioned in the operative technique for the individual disease. In some patients', an ultrasound to know the contents whether solid or liquid and bronchoscopy, may be required. The patients should be fully worked-up for general anesthesia with blood grouping and cross matching. The patients should undergo active pulmonary physiotherapy and which is continued postoperatively to hasten the recovery.

### Anesthesia

The prerequisite for any endoscopic procedure is to obtain the adequate space to work. In thoracic cage the only way to achieve this is by collapsing the ipsilateral lung. There are many different techniques to collapse the ipsilateral lung, including placement of endotracheal tube in the contralateral main stem bronchus, use of double lumen tube, double lumen endobronchial tubes, use of bronchial blockers[3] and use of low pressure CO_2_ insufflations. The optimal technique for each patient must be decided in conjunction with the team of pediatric anesthetists.

In adults few straightforward procedures, such as lung biopsy or treatment of pneumothorax can be performed under regional anesthesia. However, in children most of the procedures needs general anesthesia.

Single lung ventilation facilitates many technically demanding procedures, especially when mediastinal dissection is required. A more stable operative field is achieved by collapsing the ipsilateral lung with resultant absence of movement with ventilation. It is easy to identify isolated pulmonary nodules in a collapsed lung; however identification of air leaks becomes difficult in patients with spontaneous pneumothorax.

The application of general anesthesia using single lung ventilation may be technically difficult in small children. The smallest double-lumen device is suitable for a child with a body weight of at least 30 to 35 kilograms. This limitation precludes use of this method in smaller children, usually less than 12 years of age. In these cases, alternative techniques have to be adapted to create an adequate pleural space for endoscopic surgery. Selective intentional intubation of main stem bronchus with a cuffed or uncuffed tube can be used in small children. However, it is often difficult to achieve complete collapse of the ipsilateral lung due to presence of leak around the cuff.

Insufflation of carbon dioxide under positive pressure can result in hemodynamic compromise and is not well tolerated by small infants. High pressure in the thorax decreases cardiac index, mean arterial pressure and left ventricular stroke index and increases pulmonary artery and central venous pressures.[4] However, in very small infants this may be the only suitable technique and the trick is to use low pressure insufflation at the pressure of 5 cm of water and flow at 0.5 to 1.0 Liter/minute. The patient would get adapted to this pressure in 10–15 minutes. If there is any problem then insufflation should be discontinued for a minute and then re-insufflate.

In small children, an intraoperative endobronchial blockage can be carried out by using a balloon tipped; vascular catheter (Fogarty catheter) to achieve the selective single lung ventilation.[1] The Fogarty catheter followed by an endotracheal tube is placed into the trachea. The endotracheal tube is connected to the angled swivel valve through which the flexible bronchoscope is placed to guide the Fogarty catheter into the ipsilateral main stem bronchus. The inflation of the balloon of the Fogarty catheter would lead to collapse of the ipsilateral lung.

Any one of the above methods can be used to achieve the selective ventilation as a pre-requisite to perform VATS.

### Surgical technique

All standard equipment for endoscopic surgery, including the camera, light source, insufflator, medical grade monitor, diathermy etc. are essential to safely perform advanced VATS. For VATS in children, 5 mm and 10 mm Hopkins rod-lens telescopes are used. In small infants, 3.5 mm or the new 14-gauge endoscopic telescope may be useful. For VATS in empyema use of a hydro-laparoscope with channel to irrigate the lens is useful to keep the lens clean and decreases the operative time. The standard trocars used in laparoscopy (10, 5 and 3 mm) can be used; however short trocars without valves are specifically designed for VATS. The standard hand instruments used in laparoscopy (5 mm/3 mm) can be used; however special instruments have been designed for VATS, which can be inserted through an open incision and they have, a gentle “S” shaped curve to adapt to the shape of the thoracic cage. The theatre set-up and operative technique would depend upon the procedure being performed.

### Advantages of video assisted thoracic surgery (VATS)

Conventional thoracotomy involves extensive muscle cutting and ribs spreading, which leads to significant postoperative pain. In adults chronic pain has been reported in 50% of the cases undergoing conventional thoracotomy.[5,6] To avoid such complications various muscle sparing techniques has been developed over the years, but none these techniques even come close to the VATS procedure, where a miniscule portion of the muscle is damaged and produces less pain.[7–9] Khan and colleagues reported the muscle sparing incisions may preserve muscle strength but may not prevent chronic pain which is largely related to the spreading of the ribs.[10]

A major advantage of the VATS procedure over the conventional open thoracotomy is the potential reduction in musculoskeletal squealae. Jaureguizar[11] reported long-term follow-up of 89 children who underwent open thoracotomy for tracheaoesophageal fistula repair. Twenty one (23.6%) of these had winged scapula, eighteen (20.2%) had asymmetry of the thoracic wall due to underlying atrophy of the serratus anterior muscle and sixteen (17.6%) of them had severe scoliosis. One girl out the eleven girls operated required mammary reconstructive surgery.

The superior and magnified visualization of the entire thoracic cavity is extremely useful particularly in operating tracheaoesophageal fistula repair where the fistula is seen very clearly. The perceived disadvantage of this procedure is the violation of pleural space in those cases which can be approached extra pluerally. However, the final outcome of transplural and extra pleural approach is comparable, thus minimizing the impact of the pleural space violation.[12]

The VATS has a definite learning curve. Most of the large series have reported superior results of VATS over the open thoracotomy and have well-equipped theaters with an experienced team who regularly undertakes such procedures. Apart from the surgical challenges and the anesthesia and the intraop and the postoperative physiological alterations post a significant challenges to the success of VATS.

### Decortications for empyema thoracis

American Thoracic Society classifies the empyema formation into three stages, i.e. exudative, fibrinopurulent and organizing stage.[13] In exudative stage; there is an immediate response with outpouring of thin fluid with a low cellular content, which can be easily aspirated with needle. The pleura and the lung are mobile and the pleural fluid pH, glucose and LDH levels are all normal. This stage usually lasts for 24–72h. The fibrinopurulent stage is characterized by large quantities of pus posteriorly and laterally, with great numbers of polymorphs, nuclear leucocytes and fibrin. As the fluid thickens, loculations begins and the lung progressively becomes less expandable. The pH and glucose values show a fall with increase in LDH levels. This stage usually lasts for 7–10 days but may last for several weeks. The organizing stage is characterized by growth of fibroblast into the exudate on both the visceral and parietal pleural surfaces, producing a membranous “peel”, with increasing fibrosis, the ET becomes chronic and the lung is entrapped with inability to expand. Early intervention of ET prevents chronicity of the disease.

In the exudative stage, the management with intercostal drain is adequate. However, in the fibrino-purulent stage, ICD is not adequate to evacuate empyema completely and VATS should be performed. The advantage of VATS in empyema thoracis includes determination of the stage of the disease, breaking of all loculi with complete evacuation of thick pus and fibrinopurulant material, reduction in the bacterial load in early stage and ICD insertion in the proper dependant position. In addition, VATS gives visual impression of the condition of underlying lung, its capacity to expand and presence, site and size of BPF. As thick pus and fibrinopurulant material is removed thoroughly, the fever resolves quickly (usually 48–72h), ICD is required for short duration (usually 48–72h) and post procedure hospitalization can be reduced to 6–7 days.[13–14] The trauma of insertion of ICD under local anesthesia is avoided. The disadvantages of VATS in empyema thoracis include need of general anesthesia, operating room facilities with availability of video camera and team of experienced anesthetists and endoscopic surgeon. Some of the patients undergoing VATS are very sick and they may need post procedure careful monitoring. The VATS in ET can be either performed primarily or secondarily after failure of conventional management with intercostal drainage and antibiotics. The authors recommends conservative management for patients who present early (< 7 days), primary VATS for those who present late (>7 days) or have definite loculations on imaging studies and an open surgery for chronic disease. The patients in whom ET fails to resolve conservatively in 72h as decided on X-ray and USG of chest should undergo secondary VATS. The patients having broncho-pleural fistula can be managed conservatively provided they have good expansion of lung with ICD. The approach has been successful to decrease the morbidity and mortality from the disease.

### Pulmonary parenchymal biopsy

Lung biopsies for either diffuse or localized processes are common indications for thoracoscopy. In patients with diffuse processes, preoperative imaging with postero-lateral and lateral chest roentgenograms is usually sufficient. In patients with localized disease processes, preoperative imaging with high resolution CT scans is useful.

Thoracoscopy from the right side presents more pulmonary edges to biopsy and is preferred, unless there are specific indications not to do so. Prolonged air leaks from the biopsy site are the commonest complication. Simple sutures or ligatures often give way resulting in a broncho-pleural fistula. This is because many of these patients are subjected to high-pressure mechanical ventilation at the time of biopsy. An Endo-GIA stapling device can reduce this complication rate, but does add to the cost. This stapler requires a 12 mm port for its use and at least 5 cm of the end of the instrument must be in the chest to allow the jaws to open properly. This limits its usefulness in younger children. The 12 mm port for the stapler is placed as caudally as possible to allow the jaws to open properly. Another 5 mm/10 mm port is needed for the telescope and occasionally a third 3 mm/5 mm port may be required for retraction or dissection. Usually several biopsy specimens are obtained from these patients, often including pieces of different lobes.

Pulmonary nodules deeper than 1cm under the visceral pleura may be difficult to identify by thoracoscopy. Ipsilateral lung collapse may help or alternatively a finger can be introduced through one of the port site openings for direct tactile perception. Other options available in such cases are stereotactic needle localization, preoperative tattooing with Methylene blue, India ink or patients own blood, intra-operative ultrasonography.[15] In case malignancy is suspected, it is important to deliver the specimen in a bag, to avoid port site seeding. In most cases, the specimen is delivered at the 12 mm port site, but occasionally the port site opening may have to be extended.

Thoracoscopic sampling of pulmonary tissue gives a very high yield and results are encouraging.[16] virtually all failures of this technique relate to poor patient selection and inability to visualize the lesion, which emphasizes the role of preoperative evaluation and work-up.

### Mediastinal disease

Mediastinal lymphnodes biopsy and excision of other mediastinal lesions can be performed easily. Preoperative CT scans are of vital importance to plan the procedures. It is mandatory to have selective ventilation of the contralateral lung with collapse of the ipsilateral lung. Positioning of patients and of port sites is individualized carefully, depending on the relation of the lesion with the surrounding structures. Lateral decubitus with an anterior or posterior tilt will help move the lung out of the way by gravitational pull. Most procedures are done with two or three ports. Extensive surface biopsy specimens can be done using cup forceps, while deeper tissue can be obtained with a transthoracic, visually guided, true-cut needle.

Thymus gland is located in the superior mediastinum and its excision is indicated in some of the patients with Myasthenia Gravis. The techniques of VATS can be safely applied in these patients to decrease the morbidity of the thoracotomy.[17]

### Tracheoesophageal fistula (TEF) with Oesphageal atresia (OA)

The rapid advancement of minimal access surgical techniques and equipment has enabled the surgeon to perform TEF surgery safely in expert hands. The technique involves placing the child in 45-degree right lateral position. This allows the lungs to fall away from the mediastinum. Normally three cannulas are used. Often a fourth one is used to retract the lung. Low flow CO2 (0.5–1 liters/min, 4–6 mm Hg) is used for effecting the collapse of the ipsilateral lung. Recently two Fogarty vascular catheters have been used to block the fistula and the right lung resulting in collapse of the right lung.[18] The fistula is either ligated or clipped. The esophageal ends are anastomosed end to end extracorporeally. Intracorporeal knotting is being increasingly used as this saves precious operating time.[19]

The results of the open procedure has reached such a level of perfection that it was natural to scrutinize each and every aspect of thoracoscopic TEF repair. The published report of the open thoracotomy repairs and that of the thoracoscopic repairs has been analyzed and the results of the thoracoscopic repair are marginally better.[19]

The magnified and high detail images of the entire thoracic cavity improve the overall surgical procedure. The fistula can be visualized perpendicularly and its attachment to membranous trachea can be well visualized and the clipping site can be chosen. The suture ligation of fistula can be performed well to reduce the incidence of refistulization.[19] Biosynthetic mesh can be wrapped around the disconnected fistula to reduce refistulization.[20]

The main drawback of this procedure is that it violates the pleural cavity. However, the published data shows no adverse impact of the transplueral thoracoscopic approach over the extraplueral approach of thoracotomy. Recently successful extraplueral repair has been reported.[21]

It should be noted that all the published data is from the centers that are well equipped and are doing laparoscopy and thoracoscopy for past 10–12 years. The procedure has steep learning curve for both, the surgeon and the anesthetist. The procedure is difficult in small babies less than 2 kgs in weight and in children with significant lung disease. The technique is being gradually refined and procedural advancements are being devised. Thoracoscopic repair of TEF may become the preferred modality in future.

### Diaphragmatic eventeration and hernia

Surgical repair of the congenital diaphragmatic defects like Bochdalek's hernia and Foramen of Morgagni's hernia are being increasingly accomplished thoracoscopically in infants and neonates. Though the procedure can be done laparoscopically, thoracoscopy offers definite advantages. The working space is free of bowel and liver consequently injuries to them are minimized. Postoperative adhesive intestional obstructions are less. The extent of hyper-carbia is more in thoracoscopy.[22]

The selection of babies is of paramount importance in attempting a thoracoscopic repair of CDH. Anatomically the stomach should be in the abdomen as documented on contrast radiography. Physiologically the patients should require minimal or no ventillatory support. Peak inspiratory pressure should be less than 20 cm of water. There should not be any evidence of pulmonary hypertension.[22]

The procedure is performed using three ports; two 3 mm and one 5 mm port for telescope. The abdominal contents are reduced into abdomen by CO_2_ insufflation at the pressure of 5 mm Hg.[23] The reduction may be difficult in incarcerated hernias and when the content is liver. The contents can be reduced by the laparoscope and the defect can be repaired thoracoscopically.[22–23] The defect is closed with simple sutures using non absorbable sutures. The posterior sutures are passed around the costal cartilages and tied extracoporeally. The procedure is increasingly being completed intracorporeally. Pleggeted sutures can be used intracorporeally to close posterior margin of the defect. Large defects have to be closed by installing a mesh to close the defect.[22–23]

The procedure is being increasingly done in small children, infant and select neonates with comparable results.

### Other conditions

The division of PDA can be performed using VATS.[24] The advantage is under vision clipping of PDA without any recurrence. Video-assisted thoracoscopic surgery is superior to other techniques of ductal closure; it is also simple, rapid, cost-effective and more comfortable for the patients, in addition offering cosmetic benefits. The interventional procedure to block the PDA can be performed but needs special equipment and personnel. And in addition there is some risk of recanalization and hence many cardiologists prefer Clipping of PDA using VATS for their patients. Similarly it is possible to clip and divide the ligamentum of the vascular ring using VATS. Spontaneous pneumothorax in young adults is caused by apical bullae and primary VATS with the stapling can be performed to prevent the recurrence and decrease the morbidity of the disease.[25] In the bullae less than 2 cm in size even needloscopic ablation using laser can be safely performed.[26] With rapid advancements in the diathermy and other advanced instruments video endoscopic excision of pulmonary cysts, pulmonary sequestrations, bronchogenic cysts and pulmonary lobectomies are being increasingly performed safely. Esophageal myotomy for Achalasia cardia is safely done by VATS. The right adrenal lesions have been resected trans-diaphragmatically. Benign esophageal tumors like leiomyoma cab be removed using VATS with good results.[27]

## SUMMARY

VATS is a useful for the diagnosis and treatment of many intrathoracic conditions for which in the past, conventional thoracotomy was performed. The same results can be achieved without thoracotomy with less surgical trauma, reduced hospitalization and speedier return to normal physical activities.
